# A secure and efficient image encryption scheme based on chaotic systems and nonlinear transformations

**DOI:** 10.1038/s41598-025-15794-z

**Published:** 2025-08-25

**Authors:** Wassim Alexan, Noura H. El Shabasy, Noha Ehab, Engy Aly Maher

**Affiliations:** 1 Communications Department, Faculty of Information Engineering and Technology, German University in Cairo (GUC), New Cairo, Egypt; 2 Mathematics Department, Faculty of Basic Sciences, German University in Cairo (GUC), New Cairo, Egypt

**Keywords:** Arnold’s cat map, Chaotic maps, Cryptography, Hyperchaotic systems, Image encryption, Langton’s Ant, Security analysis, Mathematics and computing, Electrical and electronic engineering

## Abstract

The exponential growth of digital imagery and the widespread adoption of automation and IoT technologies have heightened the need for robust image encryption techniques. Traditional encryption methods such as AES and DES, though effective for textual data, struggle with the high redundancy of images and real-time processing constraints. To address these challenges, this article proposes a novel multi-image encryption scheme integrating a 5D hyperchaotic system, Arnold’s Cat Map, and Langton’s Ant to achieve high security, efficiency, and resistance to attacks. The encryption process consists of four stages: (1) key generation using a 5D hyperchaotic system, (2) byte substitution using a newly designed S-box, (3) pixel scrambling via Langton’s Ant-based diffusion, and (4) transformation using Arnold’s Cat Map. The proposed method achieves a high key space of $2^{52822}$, low correlation between encrypted pixels, and fast encryption times of 0.1602s for a $256\times 256$ image, making it suitable for real-time applications. Comprehensive security analyses, including histogram analysis, correlation coefficient evaluation, entropy measurement, differential attack resistance (NPCR and UACI), and NIST randomness tests, confirm the robustness of the encryption scheme. The results demonstrate that the proposed method outperforms existing chaotic and hybrid encryption techniques in terms of security, efficiency, and resistance to cryptographic attacks.

## Introduction

The rapid proliferation of image data, driven by the widespread adoption of automation and Internet of Things (IoT) technologies, has significantly increased the volume of complex visual information requiring robust security mechanisms^1^. Currently, image security is predominantly achieved through cryptographic techniques designed to protect data transmitted over networks, primarily the Internet^2^. Image encryption refers to the transformation of a plaintext image into an unintelligible format, ensuring that only authorized recipients possessing the correct decryption key can reconstruct the original image^3^. Recent advancements in encryption algorithms have improved their efficiency, robustness, and resilience against various attacks^4^.

Traditional encryption algorithms, originally developed for securing textual data, are increasingly inadequate for image data due to its high redundancy and large size. These methods typically result in high computational overhead and insufficient diffusion and confusion properties, leading to lower randomness in the encrypted output^5,6^. A prominent example is the Advanced Encryption Standard (AES), which, despite offering strong security guarantees, demands substantial computational resources, making it unsuitable for real-time image encryption applications^5^. For example, in^7^, carrying out 3D model encryption based on hyperchaos was shown to be about five times faster than AES-256. Similarly, the Data Encryption Standard (DES) suffers from short key lengths, rendering it vulnerable to brute-force attacks^8^. Consequently, there has been a growing interest in employing chaotic systems and other advanced cryptographic frameworks to overcome the limitations of classical schemes and enhance the security of image data^5,9^.

Chaos theory has significantly influenced the field of image encryption due to the inherent characteristics of chaotic systems, such as pseudo-randomness, ergodicity, and extreme sensitivity to initial conditions^10–12^. In the context of image encryption schemes, chaotic maps are employed to perform pixel-level diffusion and permutation operations, effectively transforming the original image into an unintelligible cipher. A key advantage of such systems is that even a minute change in the initial conditions or parameters of the chaotic system results in a completely different encryption key, thereby making unauthorized decryption virtually impossible^13,14^. This ensures a high level of robustness against brute-force and differential attacks.

Chaos-based encryption schemes are particularly efficient for small-sized images, offering low encryption times suitable for lightweight applications. However, their performance tends to degrade with increasing image size, posing a limitation for real-time high-resolution image transmission^6^. Generally, chaotic systems used in encryption are categorized into one-dimensional (1D) or multi-dimensional variants. The choice between them involves a trade-off among computational complexity, encryption speed, and security strength^15,16^. One-dimensional chaotic maps are favored in real-time applications due to their simplicity and low computational overhead. In contrast, multi-dimensional chaotic systems, while more complex and computationally intensive, offer enhanced security through increased key space and better diffusion properties.

The Arnold’s Cat Map is a well-known 2D chaotic map that has been extensively employed in image encryption due to its ability to perform deterministic yet seemingly random pixel permutations^17–21^. One of its notable properties is its ability to rearrange points within a unit square without violating the image boundaries, making it particularly suitable for preserving the structural integrity of encrypted images. Like other chaotic systems, the Arnold’s Cat Map exhibits high sensitivity to initial conditions–small variations in the transformation matrix can result in drastically different permutations of the same image, thereby contributing to strong encryption characteristics.

Another unconventional yet powerful tool in image encryption is Langton’s Ant, a two-dimensional Turing machine and cellular automaton. It operates through simple, rule-based behavior, yet generates complex, emergent patterns over time, often referred to as forming a “highway”, from initially deterministic steps. This behavior is highly advantageous in image encryption, as it introduces high levels of pixel disruption and increases the statistical complexity of the cipher image^22^. Langton’s Ant is particularly valuable in scenarios where both security and computational efficiency are critical, offering a lightweight yet effective mechanism for pixel diffusion and scrambling^18^.

This article introduces a hybrid image encryption scheme that integrates multiple advanced techniques to achieve a highly non-linear, sensitive, and secure encryption process. Specifically, the proposed method, referred to as 5HSCA, is a four-stage encryption framework that leverages the unique strengths of Arnold’s Cat Map, Langton’s Ant, a five-dimensional (5D) hyperchaotic system, and a custom-designed substitution box (S-box). The scheme is designed to operate on augmented image datasets and aims to maximize confusion, diffusion, and key sensitivity while minimizing computational overhead. The main contributions of this work are summarized as follows:

- A novel, dynamically generated S-box is constructed based on the 5D hyperchaotic system, where each unique seed produces a different S-box, thereby enhancing non-linearity and strengthening resistance to algebraic and cryptanalytic attacks.
- The initial position of Langton’s Ant is dynamically determined based on the state variables of the 5D hyperchaotic system, increasing key sensitivity and unpredictability.
- The proposed encryption scheme achieves a significantly large key space of $2^{52822}$, ensuring strong resistance against brute-force attacks.
- The memory consumption for encrypting a $256 \times 256$ image is approximately 947.808 MB, reflecting the algorithm’s computational requirements.
- The algorithm demonstrates high efficiency, achieving an encryption time of 0.1602 seconds for a $256 \times 256$ image, making it suitable for real-time applications.

The remainder of this paper is organized as follows: “Related literature” section presents a review of recent image encryption techniques reported in the literature. “Foundational mathematical constructs” section introduces the mathematical foundations and preliminaries, detailing the operational principles of each component used in the proposed encryption framework. In “Proposed 5HSCA scheme” section, the complete architecture of the proposed algorithm is described, including the sequential steps involved in the encryption process. “Performance evaluation” section provides a comprehensive performance evaluation of the proposed scheme, including statistical analyses, security assessments, and comparisons with existing state-of-the-art methods. Finally, “Conclusions and future works” section summarizes the key findings, highlights the strengths of the proposed approach, and discusses possible future enhancements and directions for further research.

## Related literature

Shannon’s ideas have been implemented previously in various image encryption techniques, where the mix between confusion and diffusion is applied. The random generation of encryption keys and S-boxes has garnered enough interest in the field of image encryption^23^. This review categorizes some of the existing literature that employed mathematical constructs mainly to produce random bits to be treated as encryption keys.

Many image encryption schemes rely on chaotic systems due to their high sensitivity to initial conditions and pseudo-randomness. The authors in^24^ propose an image encryption algorithm that utilizes the Chen chaotic system and the cross-spiral transformation to generate encryption keys. The three color channels are transformed separately using spiral-based starting points and zone diffusion (to adjust pixel values) to enhance security. Their results indicate a high entropy of 7.9985 bits, low correlation coefficients, and robustness against differential attacks. The key space is also sufficiently large to resist brute-force attacks. In^18^, the authors introduce a five-stage encryption approach combining Langton’s Ant, Arnold’s Cat Map, Mersenne Twister, and S-boxes. The scheme passes the NIST SP 800-22 test, confirming its high randomness, and exhibits a large key space, ensuring resistance to brute-force attacks. The work in^25^ employs the KAA map alongside multiple chaotic maps for confusion and diffusion. The encryption technique passes statistical randomness tests and is resistant to visual and differential attacks. The authors in^26^ use Arnold’s Cat Map for pixel permutations and the Henon chaotic map for diffusion. Their results indicate strong resistance to statistical attacks and high efficiency. However, the authors in^27^ improve the traditional logistic map with fuzzy triangular numbers, improving complexity and randomness. The scheme achieves a key space of $2^{160}$, ensuring strong resistance to brute-force attacks.

Although chaotic systems provide strong security, their implementations often suffer from computational complexity and predictability problems under certain conditions. Additionally, their performance in real-time encryption remains a challenge, particularly for high-resolution images. Recent research integrates chaotic maps with DNA coding, compressive sensing, and optimization algorithms to enhance security. The authors in^28^ combine the Chen chaotic system, Fourier transform, DNA-based keys, and S-box transformations. Their encryption scheme demonstrates a large key space, high entropy, and strong resistance to brute-force attacks. The work in^29^ proposes a DNA coding-based approach combined with compressive sensing and singular value decomposition (SVD). The scheme uses the Josephus problem and pseudo-random permutations to minimize pixel correlations, achieving strong resistance against attacks. The work in^30^ presents a color image encryption technique using fractional shifted Gegenbauer moments (FrMGMs) and a 2D logistic sine map. The technique involves pixel scrambling, key generation, and diffusion, resulting in a good key space, high sensitivity, and randomness. In^31^, the authors integrate a 3D fractional Henon map with discrete fractional Krawtchouk moments, optimized using Salp Swarm and Arithmetic Optimization algorithms. The model exhibits high key sensitivity, security, and efficiency, making it a promising encryption approach.

Hybrid methods significantly improve security and resistance against statistical attacks, but introduce computational overhead, making them less suitable for real-time applications. Further research is required to optimize execution speed while maintaining security. Moreover, S-boxes provide non-linearity in encryption algorithms, increasing their robustness against attacks. The authors in^32^ introduce an S-box formulation using a fractional 4D hyperchaotic system, combined with Arnold’s Cat map and the 2D Henon Map. Their approach achieves high entropy, resistance to statistical attacks, and a large key space. In^33^, the encryption model proposed was a three-stage encryption model utilizing Fibonacci matrices, Galois field S-boxes, and a memristive coupled neural network for key generation. The scheme secures medical images, achieving high PSNR, low MAE, and fast encryption times of 0.1s. The authors in^34^ introduce an encryption algorithm that employs pixel reorganization, chaotic key generation, row-column joint scrambling, and S-boxes. Their method demonstrates strong security, large key space, and high encryption speed. While S-boxes enhance non-linearity, their fixed structures can be vulnerable to algebraic attacks. Future research should focus on dynamic and adaptive S-box generation methods for improved security. Table 1 summarizes the key findings and research gaps from the reviewed encryption schemes.

**Table 1:** Comparison of image encryption techniques.

Category	Techniques Used	Strengths	Limitations
Chaos-based encryption	Tent and Bernoulli maps^15^, Arnold’s Cat Map and Langton’s ant^18^, 1D JoanS–MuraliP and 1D logical self-embedding chaos map^24^	Large key space, High randomness (NIST test), Good resistance to differential attacks	May suffer from key sensitivity and computational overhead in real-time scenarios
Hybrid approaches	Chaos + DNA Coding + Compressive Sensing^29^, Fibonacci Q-matrix + Galois Field S-box + Neural Networks^33^, Fractional Transforms + Optimization^31^	High security, Lower pixel correlation, Optimized performance through hybridization	Complex implementation, High computational cost, Some techniques lack robustness in real-world applications
S-box-based encryption	Custom S-Boxes with hyperchaotic systems^26,32^, Fourier-DNA coding + variable-base modulo operation^28^	Strong confusion-diffusion properties, Non-linearity enhances security	Performance depends on S-Box design and susceptibility to chosen-plaintext attacks
Transformation-based encryption	Fractional Shifted Moments + 2D Logistic-Sine Map^30^, Pixel Reorganization + Row-Column Scrambling^34^	Improved key sensitivity, Efficient scrambling techniques, High entropy values	May require additional diffusion steps for enhanced security
Enhanced chaotic maps	Fuzzy Logistic Map^27^, Improved Henon Map^26^	Improved complexity over traditional logistic maps, Large key space of $2^{160}$	Not always tested against all types of cryptanalysis attacks

Many image encryption schemes are computationally intensive, limiting their applicability in real-time scenarios. Recent advancements, particularly in chaos-based and hybrid techniques, offer enhanced security but often increase computational overhead. A comparative analysis of these methods is presented in Table 1, highlighting the trade-offs between security strength and efficiency.

## Foundational mathematical constructs

### Arnold’s cat map

Arnold’s Cat map is a chaotic map widely used in image encryption algorithms to permute the pixels of an image. The transformation works by randomizing the arrangement of the pixels. However, after enough iterations, the original image will be returned. The number of iterations required for this process to occur is known as Arnold’s period, which varies depending on the dimensions of the image. The transformation carried out by the Arnold’s Cat Map is expressed in the following form:

1 $$\begin{aligned} \begin{pmatrix} x_{n+1} \\ y_{n+1} \end{pmatrix} = \begin{pmatrix} 1 & 1 \\ 1 & 2 \end{pmatrix} \begin{pmatrix} x_n \\ y_n \end{pmatrix} \pmod {N}, \end{aligned}$$

where $x$ and $y$ represent the coordinates of a pixel in the image, and $N$ is the size of the image. This transformation applies matrix multiplication to stretch the *x* and *y* coordinates and then the modulus operator to reconstruct it within those bounds. These transformations are iterated over all pixel coordinates, causing the image to become increasingly scrambled.

**Fig. 1:**
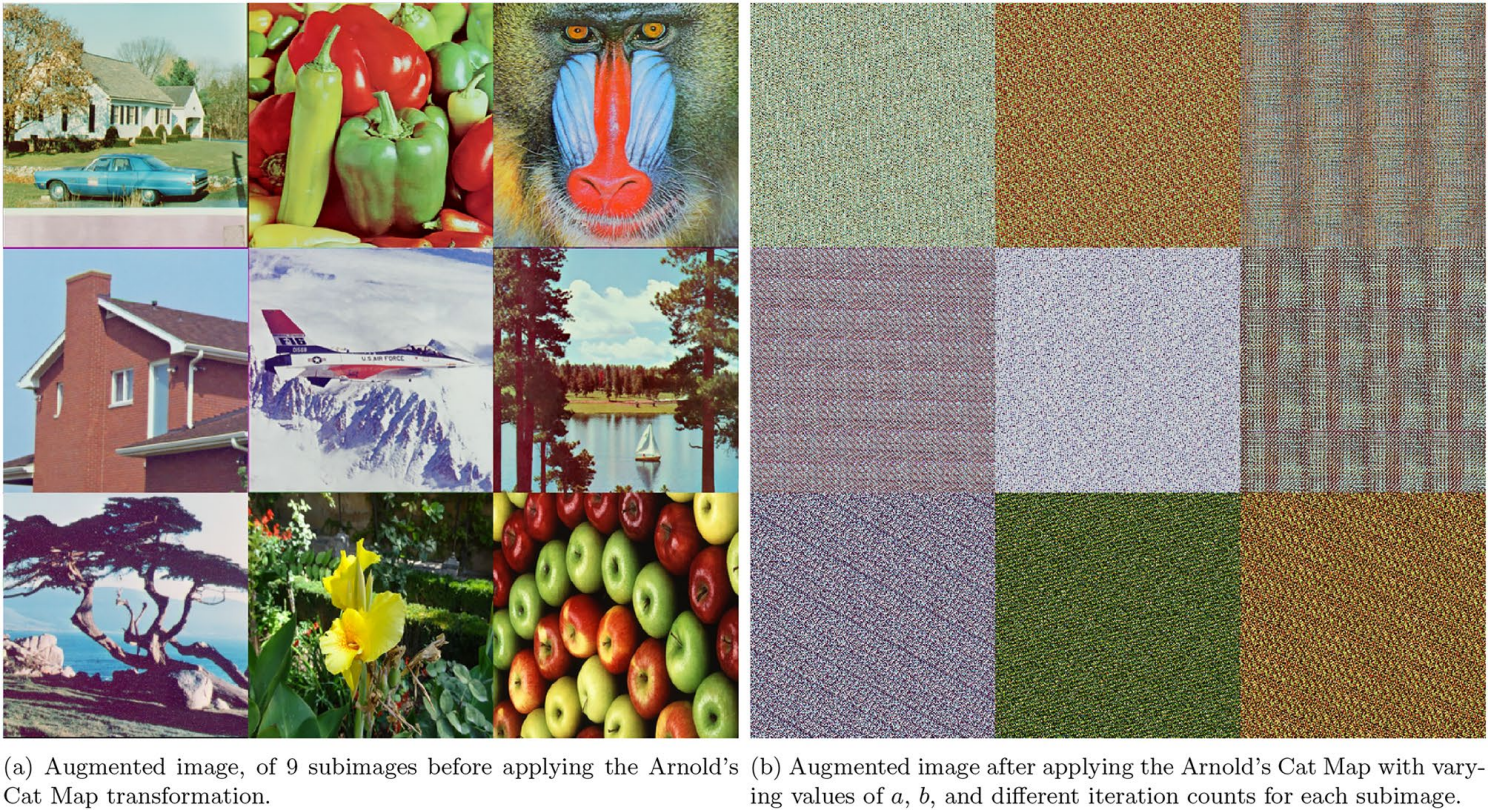
Comparison of images before and after applying the Arnold’s Cat Map transformation. Augmented image produced using the *ExampleData* and *ImageCollage* functions in Wolfram Mathematica^®^ v.13.2^35^.

In some cases, the map can be generalized by introducing the parameters $a$ and $b$ into the transformation equation:

2 $$\begin{aligned} \begin{pmatrix} x_{n+1} \\ y_{n+1} \end{pmatrix} = \begin{pmatrix} 1 & a \\ b & 1 + ab \end{pmatrix} \begin{pmatrix} x_n \\ y_n \end{pmatrix} \pmod {N}, \end{aligned}$$

where $a$ and $b$ influence the transformation, as shown in Fig. 1. Since the matrix is square, its inverse can be calculated to reverse the transformation, making decryption possible.

### Langton’s ant

**Fig. 2:**
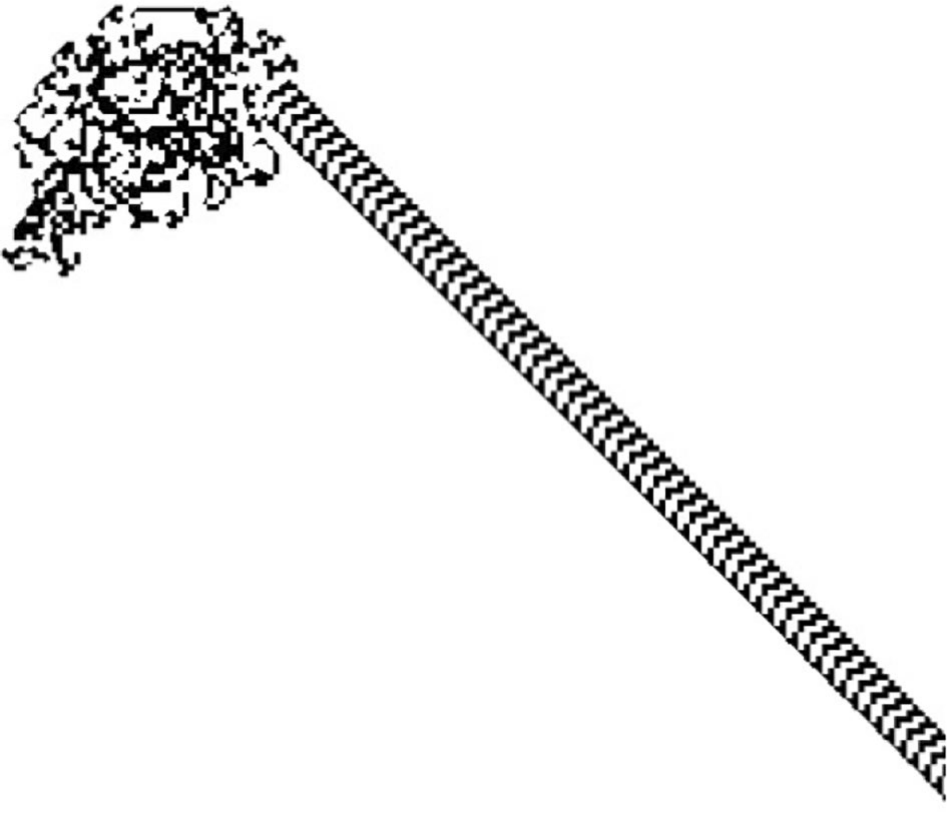
Langton’s Ant after 20, 000 iterations, starting from the center.

**Fig. 3:**
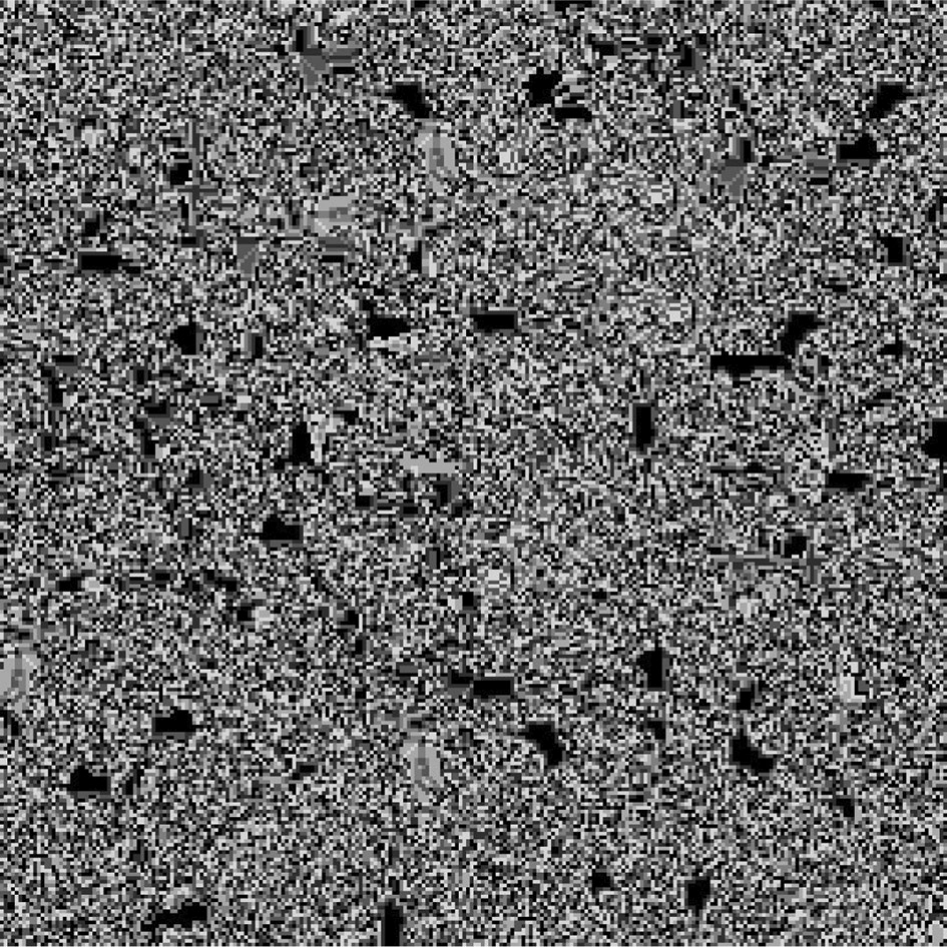
Langton’s Ant with 20, 000 iterations, using six colors and a 64-subfield grid, with one ant placed in each subfield.

Langton’s Ant is a virtual ant, which is one of the earliest models of artificial life that was first proposed by Christopher Langton in the 1980s as a basic cellular automata defined on a square grid in the manner described as follows: Each cell in the grid can be either white or black and the ant is represented by a short arrow pointing in one of the four cardinal directions (e.g. north, west, east, or south)^18^. Moving to the cell it is pointing to, the ant rotates 90 degrees to the left if the cell is white or 90 degrees to the right if it is black at each time step. Furthermore, when the ant crosses the cell, its status is inverted. Langton’s Ant, which was first created to explore the behavior of biomolecules and artificial life, shows how emergent behavior may be chaotic and evolve from simple rules. Langton’s Ant behaves unpredictably for nearly 10, 000 steps before it begins to build a periodic pattern, known as the *highway*. This pattern oscillates in a predictable manner, and in the absence of obstacles, the ant will continue to draw it forever, though with a slight drift as shown in Fig. 2. Even though the original Langton’s Ant model simply used two colors to generate patterns, adjusting the rule sets and configurations can drastically change the results. Ants’ beginning positions, the amount of colors that are triggered when they make contact with a cell, the number of ants on the field, how the ants behave when they collide, and the rules regulating edge interactions can all be changed to create variations of the ant field. Each ant’s behavior is most significantly influenced by the set of rules it follows, so the choice of effective rule sets is crucial. These rule sets will be referred to by number. Each rule can be expressed as a list of bits, where the length is equal to the number of colors, as the rule set decides whether the ant turns left or right depending on the color it encounters. Each bit indicates whether the ant turns left or right, based on the color of the tile it has stepped on. An example of a more complex ant field that uses six distinct colors rather than two, and 64 subfields instead of one with one ant in each subfield as shown in Fig. 3. By using six distinct colors instead of just two, a greater variety of rule sets can be applied, which may result in fields that are more chaotic than those generated with only two colors. The generated fields become somewhat useful as image color channel encryption keys. Theoretically, a combination of the above-stated elements should produce a sufficiently chaotic field for encrypting image color channels by expanding the color range to 256. However, expanding the color space introduces challenges, such as the emergence of non-chaotic rule sets and the tendency for fields to form somewhat homogeneous blobs near the ant’s starting position. To address these issues and generate a suitable ant field for encryption, a combination of suitable values to the factors that affect the ant’s behavior, along with a few additional techniques, is needed. Partitioning the final generated image into subfields and assigning a random number of ants to each subfield, along with assigning random starting positions for each field and introducing rule sets that handle border collision and ant-to-ant collision, are the techniques that can be applied to manage these issues. Moreover, assigning the random velocities to each ant and predetermining different rule sets for each are considered from these techniques. These ants’ rule sets are carefully chosen to maximize the intended chaotic behavior.

**Fig. 4:**
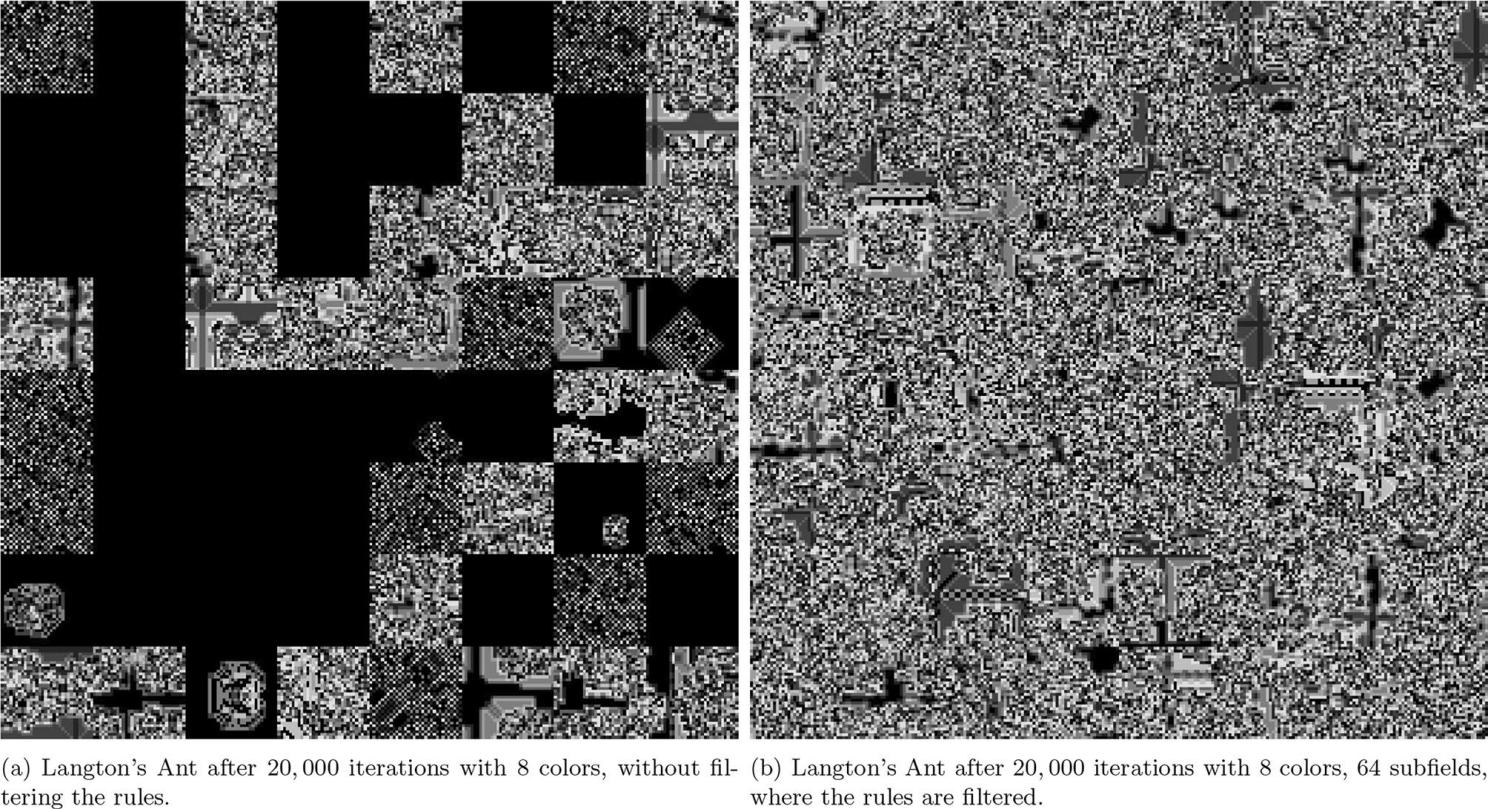
Comparison of Langton’s Ant behavior with and without rule filtering.

One of the rule sets that manages the ant-to-ant collisions, which is already used in the proposed system, can be as follows: In chess, the knights move by taking two steps ahead and then turning left. This movement scheme is applied to the ants when they collide with each other. Another rule set for managing border collision is whenever an ant collides with a border, it spawns on the opposite border, introducing a loop between the field’s borders. The problem that persists is that increasing the number of colors, and consequently the number of possible rules, also leads to a rise in the number of unreliable rules. The actual implementation of this system for encryption would require using only rules known to generate ant fields that completely fill their space. Ants may oscillate indefinitely in the middle of their fields if these rules are ignored. Increasing the number of iterations has the effect of filling some subfields while leaving others empty, as shown in Fig. 4a. The effective result of carefully choosing and applying the appropriate rules to every ant in every field is shown in Fig. 4b.

### 5D hyperchaotic system

The 5D hyperchaotic system used in this work was proposed and analyzed by the authors of^36^. The system is described by the following set of equations:

3 $$\begin{aligned} {\begin{matrix} \left\{ \begin{aligned} & \dot{x}= a (y-x),\\ & \dot{y}=cx + dy-xz+p,\\ & \dot{z}=-bz-x^2,\\ & \dot{w}=ey+fw ,\\ & \dot{p}=-rx-kp ,\\ \end{aligned} \right. \end{matrix}} \end{aligned}$$

where the variables *x*, *y*, *z*, *w* and *p* identify the system as hyperchaotic when their initial values are equal to $0.2,0.2,-0.1,-0.1$, and $-0.1$, respectively, and the system’s corresponding parameters: *a*, *b*, *c*, *d*, *e*, *f*, *r*, *k* and *q* are equal to $35,7,35,5,-0.5,-0.1,15.5,0.5$ and 0.95, respectively. The Lyapunov exponents, bifurcation, and phase diagrams of this 5D hyperchaotic system are presented in^36^, and verify that the system behaves in a hyperchaotic manner.

## Proposed 5HSCA scheme

### Encryption process

**Fig. 5:**
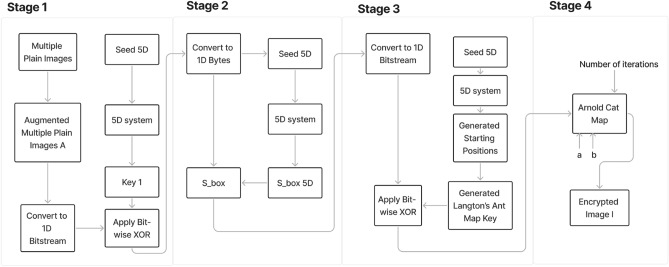
Diagram of the encryption procedure.

The encryption process shown in Fig. 5 is detailed in the following stages.

1. As an input image, a plain augmented image of dimensions $M\times N$ pixels is generated from multiple plain images and converted into a 1D bit-stream, $b_{1}$.
2. Stage 1: 5D system key.
  - The 5D system, represented in (3) is mathematically solved with certain initial values as mentioned in “Foundational mathematical constructs” section, to generate an encryption key, $k_1$. This key has the same length as the original image bit-stream.
  - $b_{1}$ is merged by bit-wise XORing with $k_1$, producing $E_{1}$.
    4 $$\begin{aligned} E_1 = b_1 \oplus k_1. \end{aligned}$$
  - $E_1$ is returned to image format.
3. Stage 2: 5D substitution box.
  - Given $Seed_{5D}$, a substitution box $Sbox_{5D}$ is constructed from the hyperchaotic system in (3), in a manner similar to that carried out in^37^. This results in an S-box as displayed in Table 2.
  - $E_1$ undergoes a byte substitution operation by applying $Sbox_{5D}$ on it, producing $E_2$.
    5 $$\begin{aligned} E_2 = Sbox_{5D} (E_1). \end{aligned}$$
  - $E_2$ is then reshaped into another bit-stream, $b_2$.
4. Stage 3: Langton’s Ant Map Application.
  - The 5D system is mathematically solved one more time to generate the ants’ starting positions in the image’s fields.
  - $b_2$ is mingled on the bit level with the ants to produce $b_3$.
    6 $$\begin{aligned} b_3 = b_2 \oplus K_{Ant}. \end{aligned}$$
5. Stage 4: Arnold Cat Application.
  - Three variables are needed to form the output of the Arnold’s Cat Map transformation. The variables are *a*, *b* and the number of iterations.
  - With these variables mentioned above, the bits in $b_3$ are converted into $b_4$ by applying the Arnold’s Cat Map repeatedly with the appropriate matrix for transformation.
  - $b_4$ is then converted to bytes to produce the final encrypted image $E_4$.

**Table 2:** An example S-box created from the 5D hyperchaotic system in (3).

231	111	56	136	8	193	11	233	62	202	154	60	96	159	80	72
103	131	23	22	44	10	54	141	235	177	14	93	153	216	181	13
124	41	59	221	175	9	12	91	164	244	38	151	187	47	30	77
147	16	239	63	172	32	243	108	36	75	163	67	37	4	115	222
146	53	98	206	167	66	18	171	105	126	228	123	21	186	189	224
144	73	204	70	82	71	195	24	102	132	190	253	133	192	137	217
42	88	78	223	120	249	3	74	157	142	139	117	85	116	119	240
209	161	43	19	40	46	155	104	194	196	156	1	135	170	143	49
205	17	241	97	87	169	213	61	113	174	6	138	57	184	25	28
210	94	160	129	48	215	166	101	134	52	246	178	208	27	58	162
34	83	33	79	234	182	230	218	220	152	198	248	238	245	107	168
76	197	39	203	130	15	183	95	68	0	26	149	127	229	226	242
100	191	64	236	31	158	106	65	86	237	145	250	51	2	179	219
69	114	188	109	118	150	92	252	112	35	50	199	214	81	227	55
185	211	201	7	225	251	99	122	29	165	176	254	140	121	180	128
173	207	212	84	5	255	110	200	45	125	90	232	247	148	89	20

### Decryption process

**Fig. 6:**
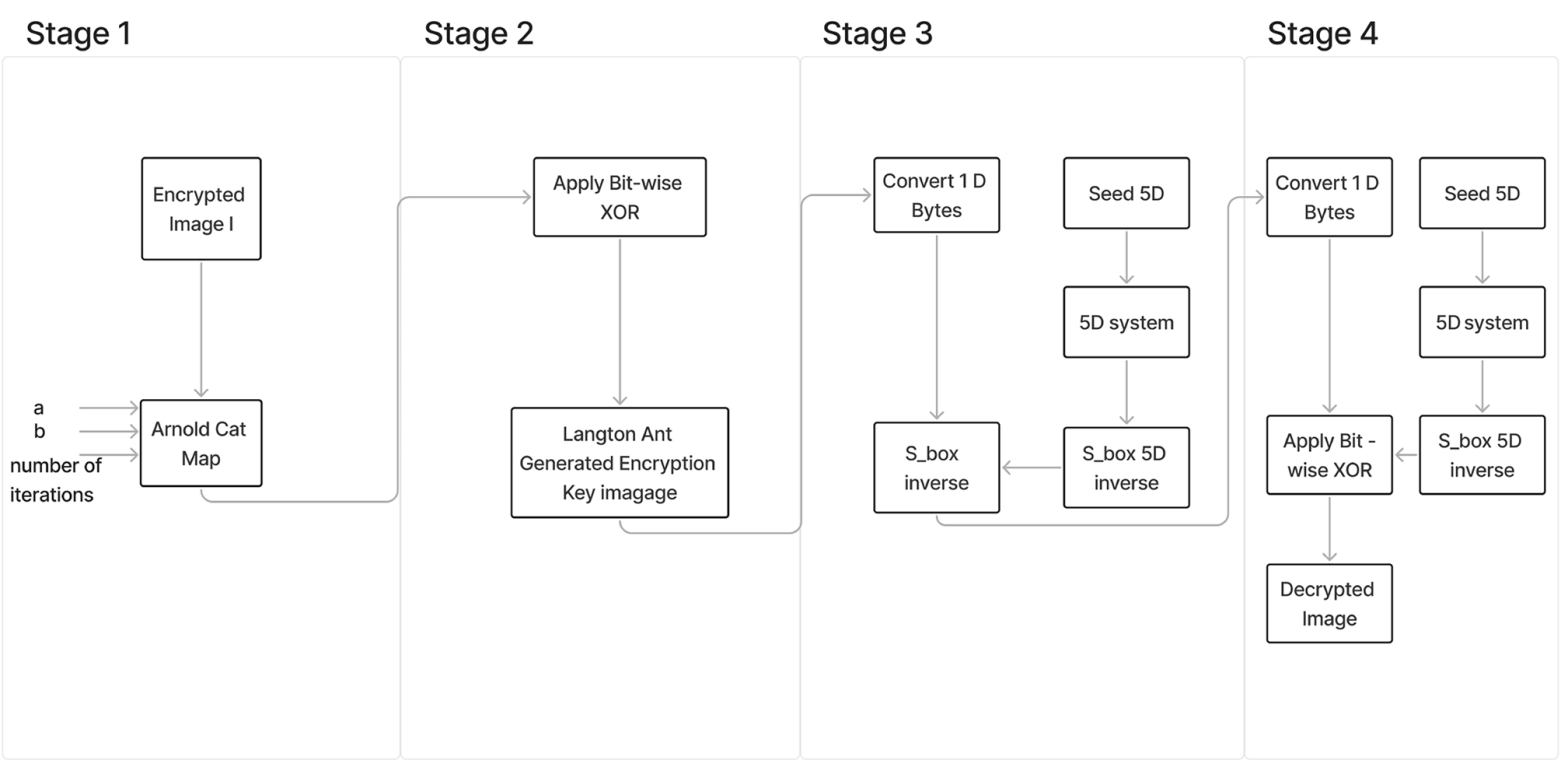
Diagram of the decryption procedure.

In this section, the decryption process, shown in Fig. 6, is described as follows.

1. Stage 4: Arnold’s Cat Map Application.
  - The encrypted image bits $b_4$ are transformed back with the help of the three variables and the Arnold’s Cat Map matrix to produce $b_3$.
2. Stage 3: Langton’s Ant Map.
  - $b_3$ is then bit-wise XORed with the Langton’s Ants key to reproduce $b_2$.
    7 $$\begin{aligned} b_2 = b_3 \oplus K_{Ant}. \end{aligned}$$
  - The 1D bit-stream $b_2$ is converted to bytes, $E_2$.
3. Stage 2: 5D substitution box.
  - The image bytes generated from stage 3, $E_2$ are permuted using an inverse S-box, $Sbox^{-1}_{5D}$ to produce $E_1$.
    8 $$\begin{aligned} E_1 = Sbox^{-1}_{5D}(E_2). \end{aligned}$$
4. Stage 1: 5D hyperchaotic system.
  - The decrypted image $E_1$ is converted into a bit-stream.
  - $E_1$ is XORed with the key generated from the 5D hyperchaotic system, $k_1$ to produce the previously generated bit-stream from the encryption part.
    9 $$\begin{aligned} b_1 = E_1 \oplus k_1. \end{aligned}$$
  - $b_1$ is then converted to bytes to produce the decrypted augmented image.

## Performance evaluation

This section carries out a comprehensive performance analysis on the proposed 5HSCA scheme. The evaluation metrics used to carry out the analysis are presented in Table 3. The computer used to conduct the following study has an Intel^®^ Core^TM^ i7 processor, with 32 GB of RAM. All tests are carried out on $256 \times 256$ images, unless mentioned otherwise. The images used for evaluating the proposed 5HSCA scheme are all sourced from the SIPI dataset^38^. Software implementation and testing of the proposed 5HSCA scheme is carried out in the Wolfram Mathematica^®^ version 13.2.

**Table 3:** Mathematical definitions of the performance assessment metrics.

Metric	Descriptive equation(s)
SSIM	$$SSIM(f,g) = l(f,g)^\alpha \cdot c(f,g)^\beta \cdot s(f,g)^\gamma$$ where $$l(f,g) = \frac{{2\mu _{x} \mu _{y} + C_{1} }}{{\mu _{x}^{2} + \mu _{y}^{2} + C_{1} }},$$$$c(f,g) = \frac{{2\sigma _{x} \sigma _{y} + C_{2} }}{{\sigma _{x}^{2} + \sigma _{y}^{2} + C_{2} }},$$$$s(f,g) = \frac{{\sigma _{{xy}} + C_{3} }}{{\sigma _{x} \sigma _{y} + C_{3} }}.{\text{ }}$$
MSE	$MSE = \frac{\sum _{i=0}^{M-1} \sum _{j=0}^{N-1} (I_{i,j} - I'_{i,j})^2}{M \times N}$
PSNR	$PSNR = 10 \log _{10} \left( \frac{I_{max}^2}{MSE}\right) , \quad I_{max} = 255$
MAE	$MAE = \frac{1}{M \times N} \sum _{i=0}^{M-1} \sum _{j=0}^{N-1} |P_{i,j} - E_{i,j}|$
Entropy	$H(m) = \sum _{i=1}^M p(m_i) \log _2 \frac{1}{p(m_i)}$
DFT	$F(k,l) = \sum _{i=0}^{N-1} \sum _{j=0}^{N-1} f(i,j) e^{-i 2\pi \left( \frac{ki}{N} + \frac{lj}{N}\right) }$
CC	$\rho (x,y) = \frac{\text {cov}(x,y)}{\sqrt{\sigma (x)} \sqrt{\sigma (y)}}, \quad \text {cov}(x,y) = \frac{1}{N} \sum _{i=1}^N (x_i - \mu _x)(y_i - \mu _y)$
NPCR	NPCR = $\frac{{\sum\nolimits_{{x = 1}}^{M} {\sum\limits_{{y = 1}}^{N} D } (x,y)}}{{M \times N}} \times 100,$ where $$D(x,y) = {\left\{ \begin{array}{ll} 0, & I(x,y) = I'(x,y) \\ 1, & \text {otherwise} \end{array}\right. }$$
UACI	$UACI = \frac{1}{M \times N} \sum _{x=1}^M \sum _{y=1}^N \frac{|I_1(x,y) - I_2(x,y)|}{255} \times 100$

### Visual and histogram analyses

**Fig. 7:**
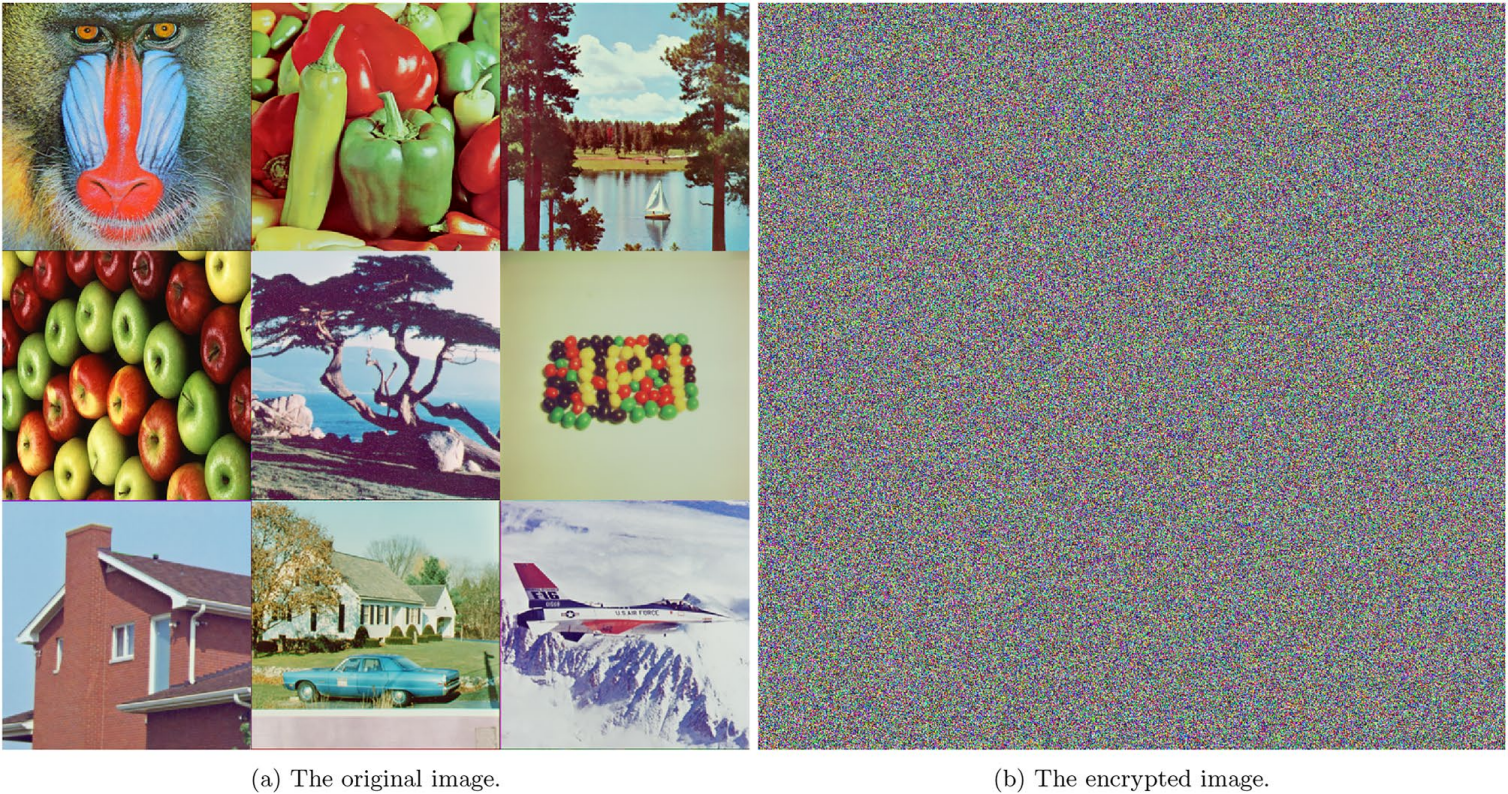
The augmented $3\times 3$ image before and after encryption. Augmented image produced using the *ExampleData* and *ImageCollage* functions in Wolfram Mathematica^®^ v.13.2^35^.

**Fig. 8:**
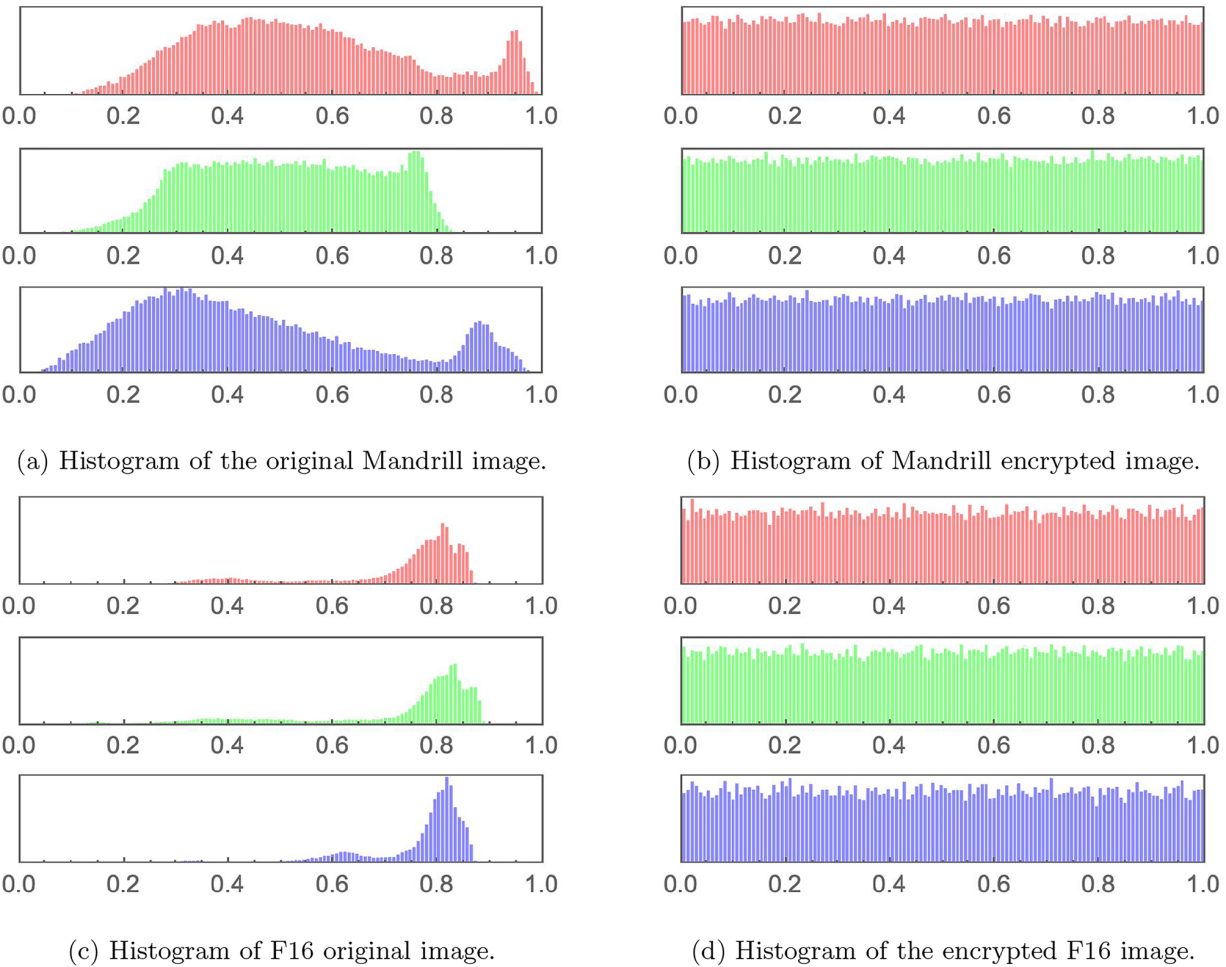
Histogram plots for samples of the original, plain images and their corresponding encrypted counterparts.

This section demonstrates the visual output generated by the proposed 5HSCA scheme. Figure 7a represents the original plain image. As shown, it displays recognizable colors, and the information is easily viewed because the pixel data is directly interpreted. In contrast, Fig. 7b displays the encrypted image after applying the proposed scheme. As seen, the pixel data has been transformed into an unreadable format, represented as a sequence of random bytes that appear as noisy patterns. This information cannot be correctly interpreted unless it is decrypted using the decryption key of the proposed 5HSCA scheme. Furthermore, histogram plots for two different image samples, Mandrill and F16, are displayed in Fig. 8. Histogram plots represent the pixel intensity distribution. As seen in Fig. 8a,c, the histograms of the plain images show specific areas with high-intensity peaks. These peaks correspond to clusters where areas are of the same color, allowing the image to be easily reconstructed. On the other hand, after applying the 5HSCA proposed scheme, Fig. 8b,d exhibit a uniform pattern across all image areas, indicating that the pixel values have been randomized. The absence of peaks means that no clear information can be detected.

### Statistical analyses

The results in Table 4 highlight the effectiveness of the proposed 5HSCA image encryption scheme based on multiple statistical metrics. The high Mean Squared Error (MSE) and low Peak Signal-to-Noise Ratio (PSNR) values demonstrate huge changes caused by encryption, which are vital for security. Furthermore, the Mean Absolute Error (MAE) values corroborate substantial pixel-level alterations. The MSE, PSNR, and MAE values are calculated for the plain and encrypted images. At the same time, the Structural Similarity Index Measure (SSIM) remains consistently 1 for all of the test images. The SSIM values are calculated for the plain and decrypted images. A perfect similarity is indicated between the decrypted and original images, thus confirming the absence of structural distortion during decryption. The entropy metrics ($H_P$ and $H_E$) illustrate a high degree of randomness in encrypted images, with the value $H_E$ approaching the ideal maximum of eight, thereby validating the robustness of encryption. Having these metrics shows how the proposed 5HSCA scheme is reliable and secure. Table 5 compares the Pixel Correlation Coefficient (PCC) values for plain and encrypted images in three directions: horizontal (H), diagonal (D), and vertical (V). In plain images, the PCC values remain high, averaging 0.93, 0.89, and 0.92 for the H, D, and V directions, respectively. This observation demonstrates strong pixel correlation and redundancy, which are inherent characteristics of natural images. The encrypted images have low PCC values, almost zero, as shown in Table 6. The H direction has an average PCC of $-0.001$, the D direction has an average PCC of $-0.00009$, and the V direction has an average PCC of 0.0002. These findings demonstrate that the proposed 5HSCA scheme effectively randomizes pixel relationships and eliminates structural dependencies. By disrupting the inherent correlations present in the original images, the scheme effectively ensures image security.

**Table 4:** The proposed encryption scheme’s statistical analysis for various metrics.

Image	SSIM	MSE	PSNR	MAE	$H_P$	$H_E$
Mandrill	1	8313.94	8.93273	75.1586	7.69625	7.99891
Peppers	1	9819.98	8.2097	81.0019	7.19038	7.99878
F16	1	10338.3	7.98631	83.0722	6.69756	7.99509
House	1	8369.38	8.90387	75.3235	7.06863	7.99767
Sailboat	1	10061.6	0.496094	8.10412	7.71189	7.99869
Jellybeans	1	9018.85	8.57929	77.939	6.58349	7.99533
Apples	1	11990.2	7.34253	89.5152	7.63364	7.99806
Tree	1	9938.43	8.15763	81.5366	7.53709	7.99895

**Table 5:** Pixel correlation coefficients in three directions: horizontal (H), diagonal (D) and vertical (V) for plain images.

Original image	H	D	V
Mandrill	0.848778	0.750624	0.79088
Peppers	0.959422	0.930426	0.966795
F16	0.92752	0.868229	0.920783
Sailboat	0.952381	0.919872	0.950138
Tree	0.968153	0.929967	0.94515
Apples	0.965065	0.956994	0.986386
Average	0.9368865	0.892685	0.926689

**Table 6:** Pixel correlation coefficients in three directions: horizontal (H), diagonal (D) and vertical (V) for encrypted images.

Encrypted image	H	D	V
Mandrill	0.00307613	0.000809074	− 0.00521955
Peppers	− 0.00329259	− 0.00567403	0.000306954
F16	0.00046871	− 0.00129673	− 0.0015519
Sailboat	− 0.00579529	0.00357263	0.00601578
Tree	0.00122393	− 0.00043615	− 0.00441973
Apples	− 0.00213933	0.00248506	− 0.010287
Average	− 0.00107641	− 0.00009002	− 0.00025259

### Differential attacks analysis

The proposed 5HSCA scheme’s sensitivity to slight changes in the plaintext image is evaluated through differential attack analysis. Robust encryption algorithms are expected to produce significantly different ciphertexts in response to minimal variations in the input. This sensitivity is quantitatively measured using two standard metrics: the Number of Pixels Change Rate (NPCR) and the Unified Average Changing Intensity (UACI). High values of NPCR indicate a large proportion of altered pixels, while high UACI values reflect the average intensity of these changes. Both of these are indicative of strong diffusion properties.

The proposed 5HSCA scheme in this work achieves a UACI of 32.2535 and an average NPCR of 99.6063, demonstrating strong resistance to differential attacks. The detailed results are presented in Tables 7 and 8.

**Table 7:** NPCR metrics for encrypted images.

Encrypted image	NPCR value
Mandrill	99.6068
Peppers	99.6068
F16	99.6017
Sailboat	99.588
Apples	99.6282
Average	99.6063

**Table 8:** UACI metrics for encrypted images.

Encrypted image	UACI values
Mandrill	29.474
Peppers	32.0919
F16	32.5773
Sailboat	32.0207
Apples	35.104
Average	32.25358

### Discrete Fourier transform analysis

**Fig. 9:**
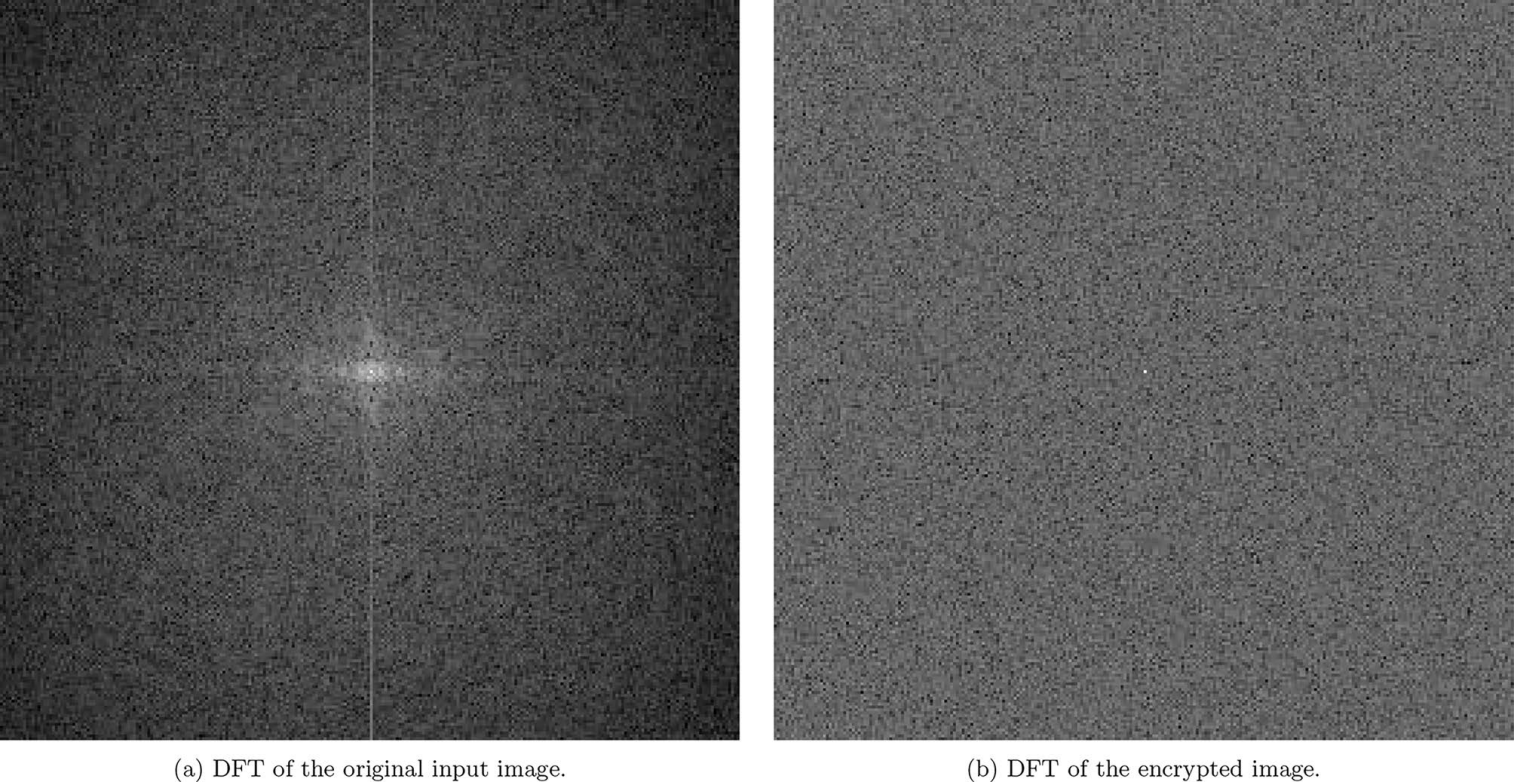
Discrete Fourier Transform applied on the Mandrill original and encrypted images.

Applying the Discrete Fourier Transform (DFT) to images before and after encryption serves as an essential tool for evaluating the robustness of the proposed 5HSCA scheme. Figure 9 illustrates the DFT analysis for the Mandrill image. The frequency representation of the plain Mandrill image, shown in Fig. 9a, reveals that low-frequency components are concentrated at the center, while higher frequencies appear as one moves outward. The relatively uniform distribution of frequencies indicates the presence of structured patterns in the original image.

In contrast, Fig. 9b presents the DFT of the encrypted image, where the frequency components appear randomly and uniformly scattered across the spectrum. The absence of any discernible symmetry or structure in the encrypted DFT confirms the effectiveness of the 5HSCA scheme in obfuscating spatial and frequency-domain features. Thereby validating its robustness against frequency-based analytical attacks.

### Complexity and time analyses

The computational efficiency of the proposed 5HSCA scheme is presented in Table 9, which reports encryption times for images of varying resolutions. The results demonstrate a direct proportionality between image size and encryption time. Specifically, an encryption time of 0.036667 seconds was recorded for a $128 \times 128$ image, while a larger $256 \times 256$ image required 0.1602 seconds.

**Table 9:** The encryption time using the proposed scheme for various image dimensions.

Single image dimension	Time (s)
$64 \times 64$	0.008425
$128 \times 128$	0.036667
$256 \times 256$	0.1602
$512 \times 512$	0.48506

**Fig. 10:**
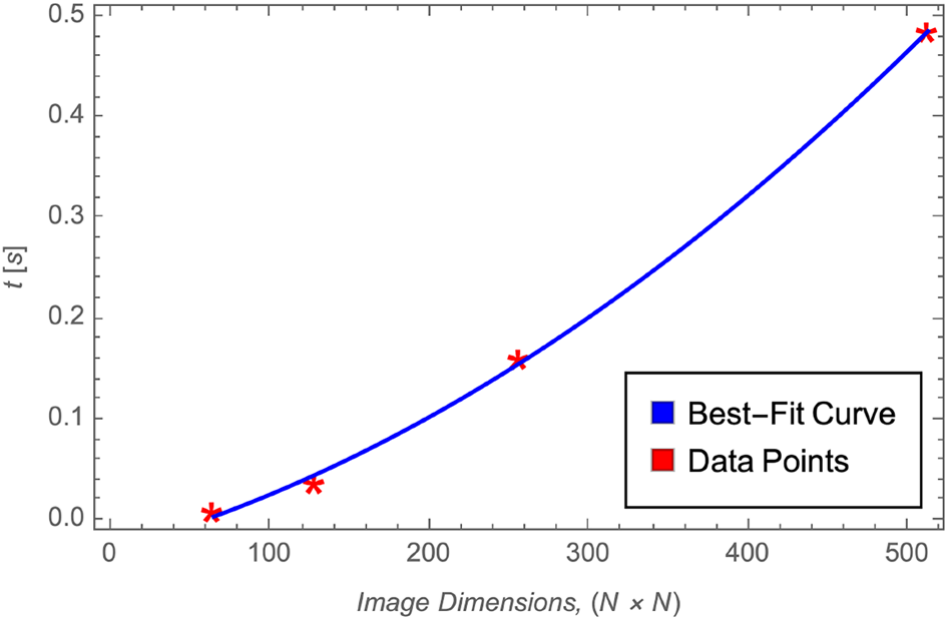
Encryption time taken, in seconds, across different image dimensions.

Furthermore, Fig. 10 illustrates the relationship between image dimensions and encryption time for the proposed 5HSCA scheme. The red asterisks denote measured encryption times for images of increasing resolution, while the blue curve represents a best-fit model. The trend indicates a quadratic growth pattern, suggesting that the encryption time scales with the image size according to a computational complexity of approximately $\mathcal {O}(N^2)$, where $N\times N$ is the image dimension. This confirms the scalability and efficiency of the 5HSCA scheme, making it suitable for high-resolution image encryption while maintaining predictable performance.

### Memory usage analysis

**Fig. 11:**
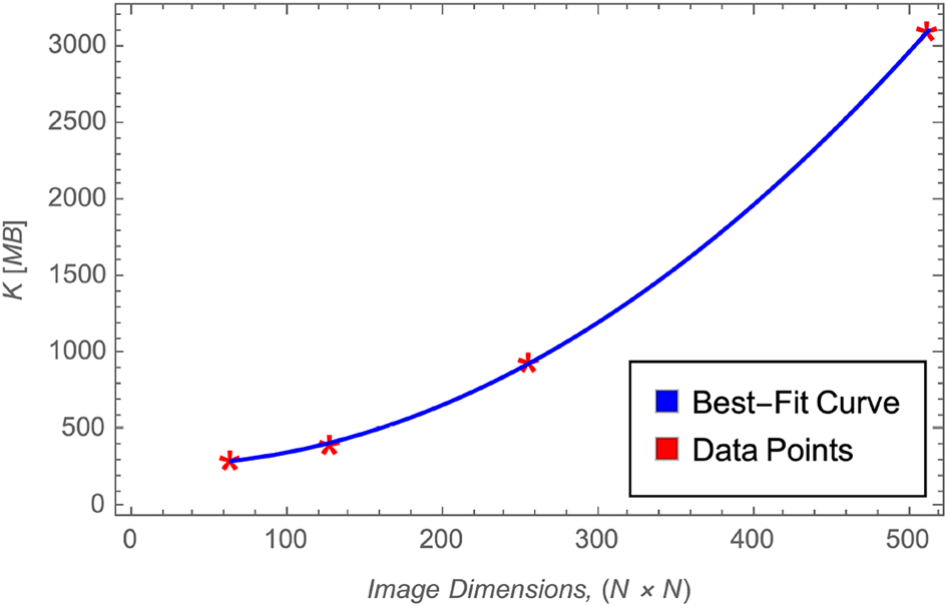
Memory used in MB across different image dimensions.

Figure 11 presents the memory consumption of the proposed 5HSCA scheme across varying image dimensions, with red markers indicating actual measurements and the blue curve representing a best-fit trend. The results demonstrate a smooth, predictable increase in memory usage as image size grows, following an approximate $\mathcal {O}(N^2)$ complexity. This is consistent with the pixel-based nature of the encryption process. Despite the increasing memory demand, the scheme remains memory-efficient, showing no abrupt spikes or overhead beyond what is expected for image data of corresponding resolution. This efficiency ensures that the 5HSCA scheme is practical for deployment on systems with constrained memory resources, including embedded and real-time imaging applications.

### S-box performance analysis

S-boxes are very important factors in applying confusion and diffusion properties in image encryption. The 5D hyperchaotic system’s PRNG is mapped to a set of integers from 0 to 255, producing the S-box matrix as shown in Table 2. This set is then analyzed based on some metrics to ensure its randomness and robustness. This evaluation involves metrics that show valuable insights into the S-box’s effectiveness, along with a detailed assessment of its suitability for achieving the desired confusion properties^39^. Below are the metrics used to evaluate the generated S-box:

1. Non-linearity (NL): It measures how changing one bit in the input can affect the output. The ideal value is 120, but normally the effective S-boxes reach values around 112.
2. Linear approximation probability (LAP): It scans the probability that the S-box is presenting a biased behavior to a particular output. The optimal value is 0.0625.
3. Differential approximation probability (DAP): It examines how the changes, extending the bit level, can affect the output. The optimal value for DAP is 0.0156.
4. Bit Independence Criterion (BIC): It reflects the relation between the encrypted output periodic patterns and the encryption steps. The ideal value is 112.
5. Strict Avalanche Criterion (SAC): It measures how the input changes affect the output. The optimal value is 0.5.

Table 10 shows the proposed S-box’s performance in comparison with the ideal values for each measurement. The table ensures the effectiveness of the generated S-box. The proposed system’s S-box achieved the ideal values in the BIC, DAP and LAP, even the SAC resulted in almost the ideal value.

**Table 10:** 5D Hyperchaotic S-box evaluation metrics compared with the ideal values.

S-box	NL	SAC	BIC	LAP	DAP
Proposed 5*D*	106	0.501465	112	0.0625	0.015625
Optimum case	112	0.5	112	0.0625	0.015625

### Occlusion and noise attack analysis

**Fig. 12:**
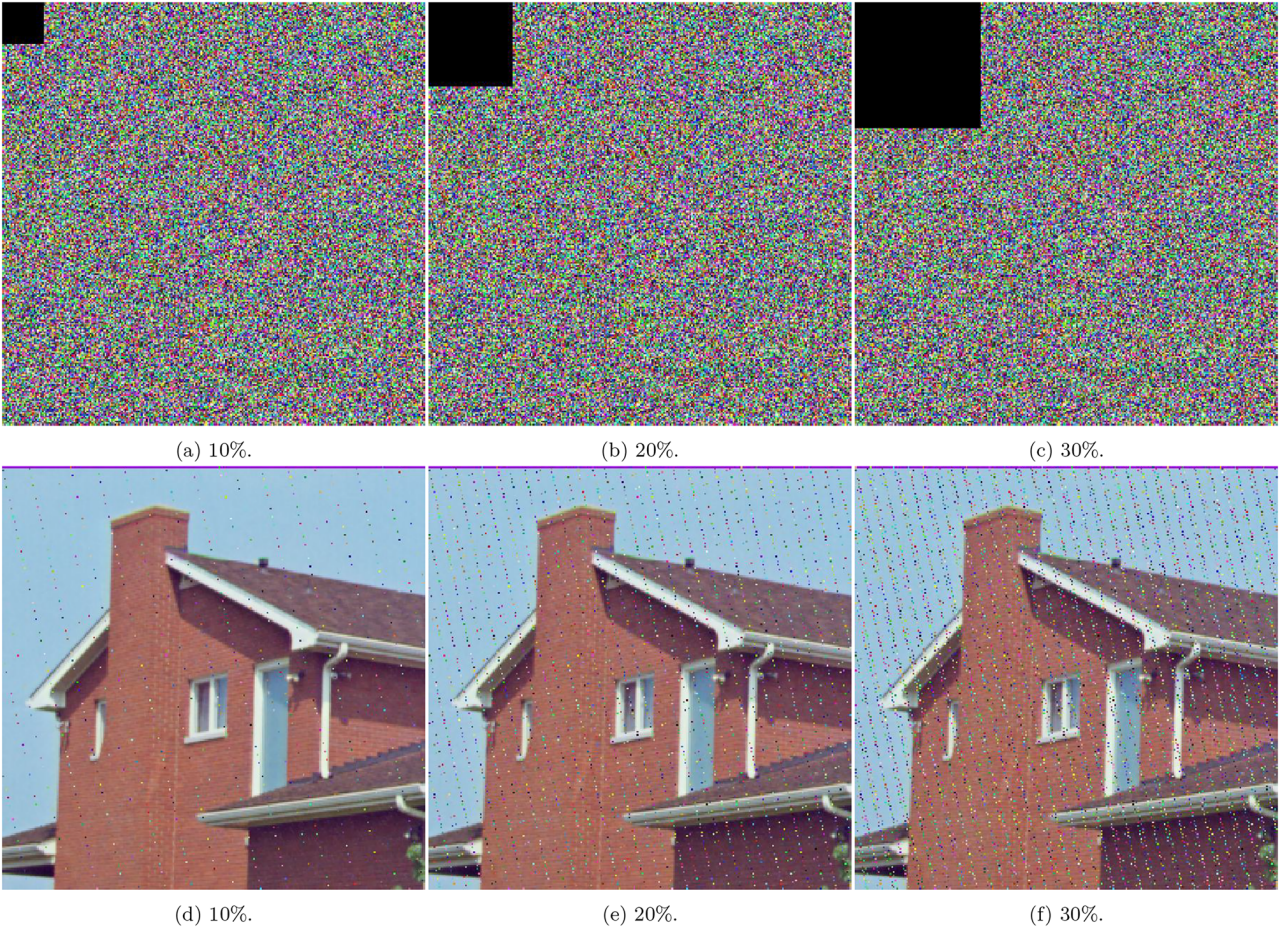
A visual showing occlusion attacks with different percentages of blocked area.

**Fig. 13:**
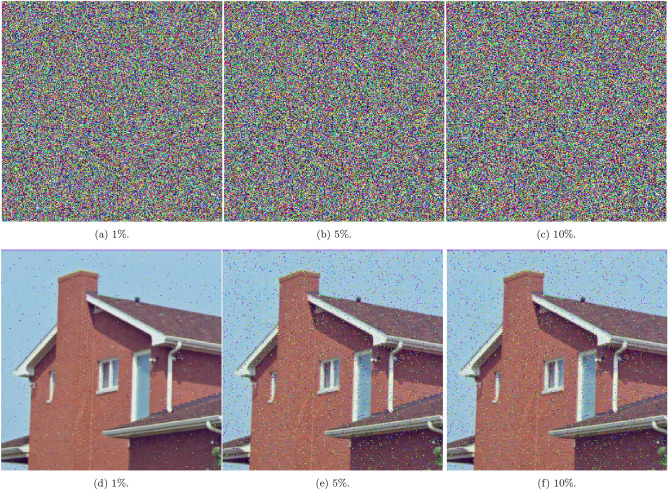
A visual showing salt-and-pepper attacks with varying levels of intensity.

**Fig. 14:**
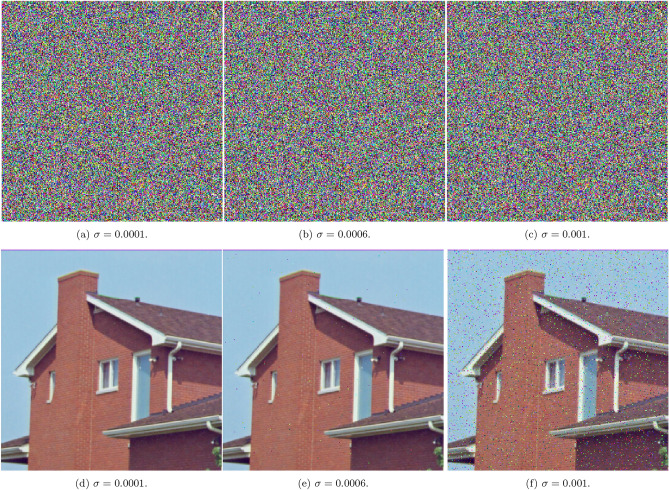
A visual showing Gaussian noise attacks with different noise standard deviation values.

This section evaluates the robustness of the proposed 5HSCA scheme under various adverse conditions, as illustrated in Figs. 12, 13, and 14.

Figure 12 investigates the impact of occlusion attacks, in which $10\%$, $20\%$, and $30\%$ of the image area are deliberately masked using black blocks. Despite the presence of significant occlusion, the corresponding decrypted images remain visually intelligible, demonstrating the scheme’s ability to accurately recover encrypted content even when parts of the ciphertext are missing.

Figure 13 analyzes the scheme’s resilience to salt-and-pepper noise, where random black and white pixels are introduced at intensities of $1\%,$ $5\%$, and $10\%$. The decrypted results retain recognizable visual content across all noise levels, indicating that the 5HSCA scheme effectively mitigates the effects of impulsive noise and ensures reliable decryption.

Figure 14 assesses the scheme’s performance under Gaussian noise with standard deviations of $\sigma = 0.0001$, 0.0006, and 0.001. Even at higher noise intensities, the decrypted images remain clearly interpretable, confirming the scheme’s robustness against signal interference and data corruption.

Table 11 quantifies the effect of noise attacks on the proposed scheme and compare it to^40^. The metrics used are PSNR and MSE, which are critical for evaluating the robustness of the encryption scheme. When dealing with occlusion attacks, the scheme shows higher distortion (MSE = 11, 101.20 at 1/4 occlusion) but still manages better noise resistance (PSNR = 6.493 at 1/2 occlusion) compared to^40^. It performs especially well against Gaussian noise, with stronger results (PSNR = 8.92697, MSE = 8, 324.98). For salt-and-pepper noise, it maintains solid performance (MSE = 12, 155.50 at 0.1 density),

In summary, these results reflect the 5HSCA scheme’s strong resistance to both occlusion and noise-based attacks, reinforcing its suitability for high-security imaging applications where data integrity, robustness, and confidentiality are critical.

**Table 11:** Noise analysis applied on different images and compared with recent literature.

Noise Type	Ratio	Metric	Proposed			^40^		
			Mandrill	Peppers	F16	Mandrill	Peppers	F16
Occlusion Attack	1/4	PSNR	7.677	7.283	6.176	10.425	10.537	10.450
		MSE	11101.20	12155.50	15685.60	6508.43	6570.40	6721.35
	1/2	PSNR	6.493	6.328	5.183	12.558	12.151	12.116
		MSE	14580.20	15144.30	19715.00	478.21	4856.10	4881.20
Gaussian Noise	0.000001	PSNR	8.92697	8.10321	7.98535	7.861	7.840	7.562
		MSE	8324.98	10063.7	10340.6	9706.50	9970.20	9991.40
	0.000007	PSNR	8.92697	8.10322	7.98535	8.052	8.082	7.852
		MSE	8324.98	10063.7	10345.2	9126.10	9117.50	9198.60
Salt and Pepper	0.01	PSNR	8.85161	8.05093	7.93526	–	–	–
		MSE	8470.71	10185.6	10460.6	–	–	–
	0.1	PSNR	7.283	7.913	7.540	–	–	–
		MSE	12155.50	10513.90	11456.60	–	–	–

### NIST SP 800-22 analysis

The NIST statistical analysis is used to evaluate the randomness of encryption schemes, based on the SP 800-22 test suite developed by the National Institute of Standards and Technology (NIST). This suite comprises a series of statistical tests designed to assess the unpredictability of binary sequences generated by cryptographic algorithms. In this study, the NIST tests were applied to a bit-stream extracted from an encrypted image produced by the proposed 5HSCA scheme.

As presented in Table 12, the 5HSCA scheme successfully passed all NIST randomness tests, with all *p*-values exceeding the significance threshold of 0.01. These results confirm the high degree of randomness in the encrypted bitstream, thereby validating the effectiveness and security of the proposed encryption method from a statistical perspective.

**Table 12:** The NIST analysis including different tests for the proposed 5HSCA scheme.

Test	*p*-value	Result
Frequency	0.487868	Success
Block Frequency	0.193022	Success
Run	0.706939	Success
Long runs of ones	0.739653	Success
Rank	0.152562	Success
Spectral F.F.T.	0.750818	Success
Non overlapping	0.999811	Success
Overlapping	0.193046	Success
Universal	0.193104	Success
Serial	0.926652	Success
Serial	0.476694	Success
Approx. entropy	0.687223	Success
Cum. sums forward	0.609749	Success
Cum. sums reverse	0.581240	Success
Random Excursions (Average)	0.462642	Success
Random Excursions Variant (Average)	0.5401975	Success

### Key space analysis

The resistance to brute-force attacks is a crucial metric to evaluate the proposed 5HSCA scheme. The key space analysis measures the number of possible encryption keys, and the higher the key space, the better the scheme. Thus, attackers will have difficulty getting the encryption key.

The proposed 5HSCA scheme has a key space of $10^{15936} \approx 2^{52822}$, calculated based on the number of variables mentioned in “Foundational mathematical constructs” section.

Arnold’s Cat Map requires 3 variables; *a*, *b* and *n* to specify the matrix. The 5D hyperchaotic system has 6 variables and 8 parameters; two sets of the values are required for initial conditions and there are 5 more variables for the S-box evaluation. Lastly, the generation of the ant fields requires a sum of 325 variables for every ant field and there are 3 fields for 3 color channels. This totals 996 variables for this proposed 5HSCA scheme. This number is multiplied by 16 to take into consideration the machine precision. The final value, 15936 is high enough to be resistant to brute-force attacks.

### Comparative analysis with the literature

Table 13 presents a comparative analysis of the proposed 5HSCA scheme against existing methods in the literature based on key performance metrics such as PSNR, MAE, entropy, PCC, NPCR, and UACI. The results demonstrate that the proposed method achieves lower PSNR and higher entropy, indicating stronger encryption with greater randomness. Additionally, the correlation coefficients for encrypted images are close to zero, confirming effective pixel decorrelation. The NPCR and UACI values exceed $99.6\%$ and $32\%$, respectively, highlighting the scheme’s strong resistance to differential attacks. Compared to existing approaches, the proposed 5HSCA scheme offers a superior balance between security and efficiency, making it a robust solution for image encryption.

**Table 13:** Comparative analysis with counterpart schemes from recent literature.

Image		PSNR	MAE	Entropy	CC			NPCR	UACI
					H	D	V		
Peppers	Proposed	8.11058	81.8345	7.99887	− 0.00329259	− 0.00567403	0.000306954	99.6068	32.0919
	^10^	8.09611	82.0162	7.99917	0.00532219	− 0.000599774	0.00445526	99.6119	32.1632
	^18^	8.06855	82.3332	–	0.00157365	0.00494262	− 0.0067555	–	–
	^24^	28.5065	–	7.9985	0.0035	− 0.0001	− 0.0042	99.611	33.471
	^25^	8.1624	81.83799	7.9976	− 0.00021	0.00027	0.00128	–	–
	^26^	–	–	7.9993	0.0017	0.0017	− 0.0026	99.4133	32.1379
	^28^	8.11813	81.9145	7.99896	− 0.00113946	0.00469114	0.00184432	99.5768	32.1233
	^29^	34.146	–	7.9971	− 0.0017	0.0064	0.0004	99.62	33.48
	^30^	8.1272	–	7.9992	− 0.000666	0.0157	− 0.004867	99.6138	33.4485
	^32^	–	–	7.9962	0.000866	− 0.0024	− 0.00206	99.6094	33.3844
	^33^	8.12053	81.8107	7.99887	− 0.000938351	0.0017651	− 0.000462341	99.6058	32.0826
	^34^	–	–	–	–	–	–	–	–
Mandrill	Proposed	8.93273	75.1586	7.9988	0.00307613	0.000809074	− 0.00521955	99.6068	29.474
	^10^	8.93217	75.0714	7.99914	0.000208593	− 0.00393718	0.000522149	99.5967	29.4397
	^18^	8.93145	75.1741	–	− 0.00286339	− 0.00280182	− 0.00290341	–	–
	^24^	24.7007	–	7.9985	0.0006	− 0.0004	0.002	99.617	33.467
	^25^	8.9283	75.46050	7.9967	− 0.00004	− 0.000021	0.00039	–	–
	^26^	–	–	7.9971	0.0030	0.0054	− 0.0045	99.3591	28.8010
	^28^	8.92843	75.20998	7.99916	− 0.00290101	− 0.00109368	0.000386728	99.6201	29.494
	^29^	–	–	7.9972	–	–	–	99.61	33.52
	^30^	–	–	–	–	–	–	–	–
	^32^	–	–	7.997	− 0.001833	− 0.000206	0.0003266	99.6099	33.3453
	^33^	8.93971	75.0375	7.99886	− 0.000382536	0.00424926	− 0.0045848	99.6201	29.4265
	^34^	–	–	7.9993	0.9227	0.8476	0.8597	99.60	33.40

Table 14 presents the evaluation metrics of the proposed hyperchaotic S-box in comparison with existing S-box designs from the literature. The proposed S-box achieves NL $= 106$ and BIC $= 112$, which are close to the optimal values, indicating strong non-linearity properties. The SAC value of 0.501465 is nearly ideal, demonstrating good avalanche characteristics. Additionally, the LAP and DAP values (0.0625 and 0.01562, respectively) align with the optimal case, confirming the S-box’s robustness against linear and differential attacks. While some existing S-boxes achieve slightly higher non-linearity, the proposed design offers a balanced trade-off between security parameters, making it a strong candidate for cryptographic applications.

**Table 14:** 5D hyperchaotic S-box evaluation metrics comparison with recent literature.

S-box	NL	SAC	BIC	LAP	DAP
Proposed 5D	106	0.501465	112	0.0625	0.01562
^4^	108	0.499634	105.333	0.0885417	0.01562
^10^ [S-box1]	108	0.506836	100	0.109375	0.015625
^10^ [S-box2]	108	0.499756	108	0.078125	0.015625
^10^ [S-box3]	108	0.498047	100	0.109375	0.015625
^18^	108	0.49414	108	0.078125	0.01562
^28^	110	0.500732	108	0.078125	0.01562
^32^	112	0.5725	103.36	0.1406	–
^33^ [S-box1]	108	0.50488	108	0.078125	0.01562
^33^ [S-box2]	108	0.507324	108	0.078125	0.01562
^33^ [S-box3]	110	0.492188	108	0.078125	0.01562
Optimum	112	0.5	112	0.0625	0.01562

Table 15 compares the key space of the 5HSCA encryption scheme with existing methods in the literature. The proposed scheme achieves an exceptionally large key space of $10^{15936}$ $(\approx 2^{52822})$, significantly surpassing other approaches, ensuring strong resistance against brute-force attacks. While some existing methods exhibit relatively large key spaces, such as $10^{15696}$ $(\approx 2^{52141})$ and $10^{1840}$ $(\approx 2^{6112})$, many others have much lower key spaces, which may be vulnerable to modern computational attacks. The substantial improvement in key space highlights the strength of the proposed hyperchaotic system, reinforcing its security advantages for cryptographic applications.

**Table 15:** Key space comparison with recent literature.

Scheme	Key space
Proposed	$10^{15936} \approx 2^{52822}$
^4^	$2^{4624}$
^10^	$2^{10524}$
^18^	$10^{15696} \approx 2^{52141}$
^25^	$10^{144} \approx 2^{478}$
^24^	$10^{142} \approx 2^{472}$
^26^	$10^{84}$
^27^	$2^{160}$
^28^	$2^{1754}$
^29^	$2^{465}$
^30^	$10^{99}$
^32^	$2^{942}$
^33^	$10^{1840} \approx 2^{6112}$
^34^	$10^{161}$

The encryption time results presented in Table 16 demonstrate the strong efficiency of the proposed algorithm, achieving a competitive runtime of 0.1602 seconds on a standard Intel^®^ Core^TM^ i7 machine with 32 GB of RAM. While the method in^4^ reports a lower per-image encryption time of 0.0163 seconds, this is actually the per-image batch efficiency (amortized across 256 images), effectively distributing the overhead and not reflecting the true cost of encrypting individual images. In contrast, our proposed scheme reports the standalone encryption time for a single image without batch amortization, ensuring a more realistic and transparent performance measure. Compared to other recent methods, the proposed algorithm demonstrates a favorable balance between speed and cryptographic strength, outperforming several approaches with higher encryption times, such as those in^18,25^, and^34^, and maintaining practical applicability for real-time image security tasks.

**Table 16:** Encryption time comparison with recent literature.

Scheme	Time [s]	Machine specifications
Proposed	0.1602	2.7 GHz Intel^®^ Core^TM^ i7, 32 GB
^4^	0.0163	2.7 GHz Intel^®^ Core^TM^ i7, 32 GB
^10^ PC A	0.148071	Intel^®^ Core^TM^ i9, 32 GB
^10^ PC B	0.230717	Intel^®^ Core^TM^ i7, 8 GB
^18^	0.427	AMD^®^ Ryzen^TM^ 5600H Mobile 3.3 GHz, 16 GB
^25^	2.750966	3.4 GHz Intel^®^ Core^TM^ i7, 8 GB
^28^	0.021658	3.3 GHz, AMD^®^ Ryzen 9, 5900 HX, 32 GB
^33^	0.0944182	AMD^®^ Ryzen^TM^ 5600H Mobile 3.3 GHz, 16 GB
^34^	0.5	N/A

## Conclusions and future works

### Conclusions

This article introduced a novel multi-image encryption scheme that leverages 5D hyperchaotic systems, Arnold’s Cat Map, and Langton’s Ant to enhance security, efficiency, and robustness against cryptographic attacks. By integrating multiple chaotic techniques, the 5HSCA scheme achieves a high key space ($2^{52822}$), low correlation between encrypted pixels, and rapid encryption times (0.1602s for $256\times 256$ images), demonstrating its practical suitability for time-sensitive scenarios.

The proposed scheme’s strong performance in security analyses, including histogram analysis, statistical randomness testing, pixel correlation evaluation, and differential attack resistance (NPCR and UACI), confirms its resilience against brute-force, statistical, and differential attacks. Compared to existing encryption methods, the 5HSCA approach offers superior confusion and diffusion properties, ensuring that even minor changes in the plaintext image produce significant variations in the ciphertext.

A novel, dynamically generated S-box is constructed based on the 5D hyperchaotic system, where each unique seed produces a different S-box, thereby enhancing non-linearity and strengthening resistance to algebraic and cryptanalytic attacks. This dynamic mechanism further contributes to the scheme’s high level of confusion and overall cryptographic strength.

Due to its efficiency, security, and adaptability, the proposed 5HSCA encryption scheme is particularly well-suited for real-time image encryption applications, such as medical diagnostics^33,41^, satellite imaging^42,43^, and other high-security domains where speed and reliability are critical^44^.

### Limitations and future research directions

Despite its strengths, one limitation of the 5HSCA encryption scheme is its computational complexity for very high-resolution images. While the encryption time is efficient for standard image sizes (e.g., 0.1602s for $256\times 256$ images), the processing time increases for larger images due to the multi-stage transformations and chaotic operations. This trade-off between security and computational efficiency is a common challenge in chaos-based encryption schemes. However, this limitation presents an opportunity for further improvement, as optimizing the algorithm for large-scale images could make it even more practical for real-world applications.

Additionally, the memory consumption of the proposed algorithm may pose constraints when deployed on embedded or resource-constrained devices, such as IoT sensors or mobile platforms. These limitations highlight the need for lightweight implementations that maintain security while reducing memory overhead. Possible optimizations include designing more compact chaotic maps, reducing the number of transformation stages, or leveraging hardware-based acceleration. In particular, implementing the algorithm using parallel processing techniques (e.g. GPU acceleration) can significantly boost performance and enable real-time encryption for higher-resolution images without compromising security^45^.

Overall, this study lays a strong foundation for developing highly secure and efficient image encryption techniques. Future advancements will focus on scalability, adaptability, and quantum security, ensuring that the proposed 5HSCA scheme remains robust and efficient amid the ever-evolving landscape of cybersecurity threats.

## Data Availability

The datasets used and/or analyzed during the current study are available from the corresponding author on reasonable request.
